# Multidrug Resistance and Clinical Predictors of Mortality in Burkholderia cepacia complex Bacteremia: Local Evidence and Global Perspectives With a Review of the Literature

**DOI:** 10.7759/cureus.97396

**Published:** 2025-11-21

**Authors:** Soumayan Mondal, Nihar Ranjan Mohanty, Sidharth S Pattnaik, Arushi Choudhary, Lovy Aggarwal, Shubhransu Patro

**Affiliations:** 1 Internal Medicine, Kalinga Institute of Medical Sciences, Bhubaneswar, IND

**Keywords:** bloodstream infections, burkholderia, burkholderia cepacia complex, clinical predictors, mortality, multidrug-resistance, systematic review

## Abstract

Background

*Burkholderia cepacia complex* (BCC) is an opportunistic, intrinsically multidrug-resistant (MDR) pathogen increasingly recognized as a cause of bloodstream infection (BSI) in non-cystic fibrosis adults. Data from South Asia remain limited, and predictors of outcomes have not been systematically evaluated using standardized MDR definitions.

Materials and methods

We conducted a six-year retrospective study (January 2019-December 2024) at a 2,000-bed tertiary care hospital in eastern India. Adults (≥18 years) with culture-confirmed BCC BSI and available antimicrobial susceptibility data were included. Antimicrobial susceptibility testing was performed using VITEK® 2 (bioMérieux, France) and interpreted according to Clinical and Laboratory Standards Institute (CLSI) M100 (33rd edition). MDR was defined as resistance to three or more of six antibiotic classes. Independent predictors of in-hospital mortality were assessed using multivariable logistic regression. The primary objective was to identify independent clinical predictors of in-hospital mortality in adults with BCC BSI. A secondary objective was to describe antimicrobial resistance patterns and contextualize findings through a Preferred Reporting Items for Systematic Reviews and Meta-Analyses (PRISMA)-guided systematic review (2004-2024).

Results

Among 124 patients (median age 48 years; 52.4% male), 64.5% of isolates were MDR. Overall in-hospital mortality was 33.9%. Carbapenem resistance was high (imipenem 61.3%, meropenem 56.5%), while minocycline (85.5% susceptible), trimethoprim-sulfamethoxazole (TMP-SMX) (52.4%), and levofloxacin (80.6%) retained the most significant activity. In multivariable analysis, a higher qSOFA score (adjusted OR 2.26, p = 0.016), ICU admission (OR 3.37, p = 0.023), and multiorgan dysfunction syndrome (OR 3.05, p = 0.050) independently predicted mortality; the MDR phenotype was not significant. The systematic review identified 14 studies (n = 1,541) with mortality ranging from 21% to 64%, with most cohorts clustering around 30-50%. High carbapenem resistance and preserved susceptibility to TMP-SMX and minocycline were consistent global findings.

Conclusions

Across local and global data, severity of illness rather than MDR phenotype was the strongest predictor of mortality in BCC bacteremia. Widespread resistance to β-lactams and carbapenems limits empiric options, whereas minocycline, TMP-SMX, and levofloxacin remain the most reliable agents. Rapid species-level diagnostics, timely susceptibility testing, and targeted antimicrobial stewardship are essential to improve clinical outcomes and infection control in high-risk hospital settings.

## Introduction

The *Burkholderia cepacia complex* (BCC) comprises at least 24 genetically distinct but phenotypically similar species of non-fermenting Gram-negative bacilli with broad environmental distribution and opportunistic pathogenic potential [1]. Described initially as phytopathogens, BCC organisms have emerged as clinically critical nosocomial pathogens, particularly among immunocompromised and critically ill adults [2]. Their capacity to persist in moist environments and resist disinfectants has led to repeated hospital outbreaks linked to contaminated intravenous solutions, antiseptics, nebulizer fluids, and medical devices [3-5].

Although BCC infection was historically associated with cystic fibrosis (CF), the past two decades have seen an increasing burden of bloodstream infections (BSIs) in non-CF populations, especially in oncology and intensive-care settings [6]. These infections are clinically significant because BCC exhibits extensive intrinsic and acquired antimicrobial resistance mediated by efflux pumps, inducible β-lactamases, and reduced membrane permeability [1,2]. Agents such as polymyxins, aminoglycosides, and aztreonam are inherently inactive, leaving limited therapeutic options. Trimethoprim-sulfamethoxazole (TMP-SMX) remains the most reliable drug, though regional resistance varies. Data from the SENTRY Antimicrobial Surveillance Program (2014-2018) demonstrated sustained susceptibility of BCC to TMP-SMX (93%) and minocycline (88%), with high resistance to carbapenems and aminoglycosides [7].

Clinical outcomes in BCC bacteremia remain poor. Reported mortality ranges from 25% to over 50% in ICU-predominant cohorts [6,8], with independent predictors including higher SOFA or SAPS II scores, renal dysfunction, and inappropriate empiric therapy [6]. Recent studies have emphasized the importance of rapid diagnostics and antimicrobial stewardship interventions, which shorten the duration of intravenous therapy and hospital stay in Gram-negative BSIs [9].

Despite the growing recognition of BCC in non-CF adults, comprehensive data from South Asia remain limited. Existing studies are essentially outbreak reports or single-year case series, and few employ standardized multidrug-resistance (MDR) definitions or evaluate outcome determinants beyond crude mortality [5,6].

The present study describes the clinical characteristics, antimicrobial resistance patterns, and outcomes of adult patients with BCC BSIs over a six-year period at a tertiary-care hospital in eastern India. In parallel, a Preferred Reporting Items for Systematic Reviews and Meta-Analyses (PRISMA)-guided systematic review was performed to contextualize local findings within global literature on BCC bacteremia.

## Materials and methods

Study design and setting

We conducted a retrospective observational study at Kalinga Institute of Medical Sciences, Bhubaneswar, India, a 2,000-bed tertiary care teaching hospital. The study included culture-confirmed BSIs caused by BCC between January 2019 and December 2024. The protocol was approved by the Institutional Ethics Committee of Kalinga Institute of Medical Sciences (approval number: KIIT/KIMS/IEC/2279/2025) and conducted in accordance with the Declaration of Helsinki.

Patient selection and data collection

All adult patients (≥18 years) with at least one positive blood culture for BCC and available antimicrobial susceptibility testing (AST) results were included. Patients with polymicrobial bacteremia or incomplete clinical/microbiological data were excluded. Demographic and clinical data, including comorbidities, severity of illness, source of infection, organ-support interventions, and in-hospital outcomes, were extracted from the electronic medical record and microbiology information system and entered into a pre-tested Excel spreadsheet (version 16.0; Microsoft Corp., Redmond, WA, USA). Data were independently verified by two investigators for completeness and accuracy.

Microbiological methods and antimicrobial susceptibility testing

Blood cultures were processed using the automated BACT/ALERT system (bioMérieux, France). Positive samples were subcultured onto blood and MacConkey agar and incubated aerobically at 35-37°C for 18-24 hours. Colonies showing non-lactose-fermenting growth with characteristic morphology were subjected to standard biochemical tests and confirmed as BCC using the VITEK® 2 GN identification system (bioMérieux, France).

AST was performed in the same laboratory using the VITEK® 2 Compact automated system, and results were interpreted according to the Clinical and Laboratory Standards Institute (CLSI) M100, 33rd edition (2023) [10]. Internal quality-control strains (*Escherichia coli* ATCC 25922 and *Pseudomonas aeruginosa* ATCC 27853) were tested weekly. Analysis was restricted to agents with known or potential clinical activity against BCC: ceftazidime, cefepime, meropenem, imipenem, piperacillin-tazobactam, cefoperazone-sulbactam, TMP-SMX, ciprofloxacin, levofloxacin, tigecycline, and minocycline. Agents to which BCC is intrinsically resistant (polymyxins, aminoglycosides, and aztreonam) were excluded to avoid overestimation of MDR prevalence [1]. Species-level identification within the BCC (e.g., *Burkholderia cenocepacia*, *Burkholderia multivorans*) was not routinely performed due to limited molecular diagnostic capacity; isolates were identified at the complex level using VITEK® 2.

Definition of multidrug resistance

MDR was defined as resistance to three or more of the following six antibiotic classes: β-lactams (ceftazidime, cefepime); β-lactam/β-lactamase inhibitor combinations (piperacillin-tazobactam, cefoperazone-sulbactam); carbapenems (meropenem, imipenem); fluoroquinolones (ciprofloxacin, levofloxacin); tetracyclines (tigecycline, minocycline); and sulfonamides (TMP-SMX). The definition followed the international consensus criteria proposed by Magiorakos et al. [11]. Intermediate or untested results were treated as susceptible to prevent artificial inflation of resistance rates, consistent with prior MDR surveillance studies [11]; this may slightly underestimate resistance prevalence and was acknowledged as a study limitation.

Clinical severity and outcomes

Severity at presentation was assessed using the quick Sequential Organ Failure Assessment (qSOFA) score. Additional indicators included the need for ICU admission, mechanical ventilation, vasopressor therapy, and presence of acute kidney injury (AKI), acute respiratory distress syndrome (ARDS), or multiorgan dysfunction syndrome (MODS), defined per KDIGO 2012 and SOFA criteria [12,13]. The primary outcome was in-hospital mortality. Secondary outcomes included ICU and total hospital length of stay, duration of organ-support therapy, and infection-source characteristics.

Systematic literature review

**Figure 1:**
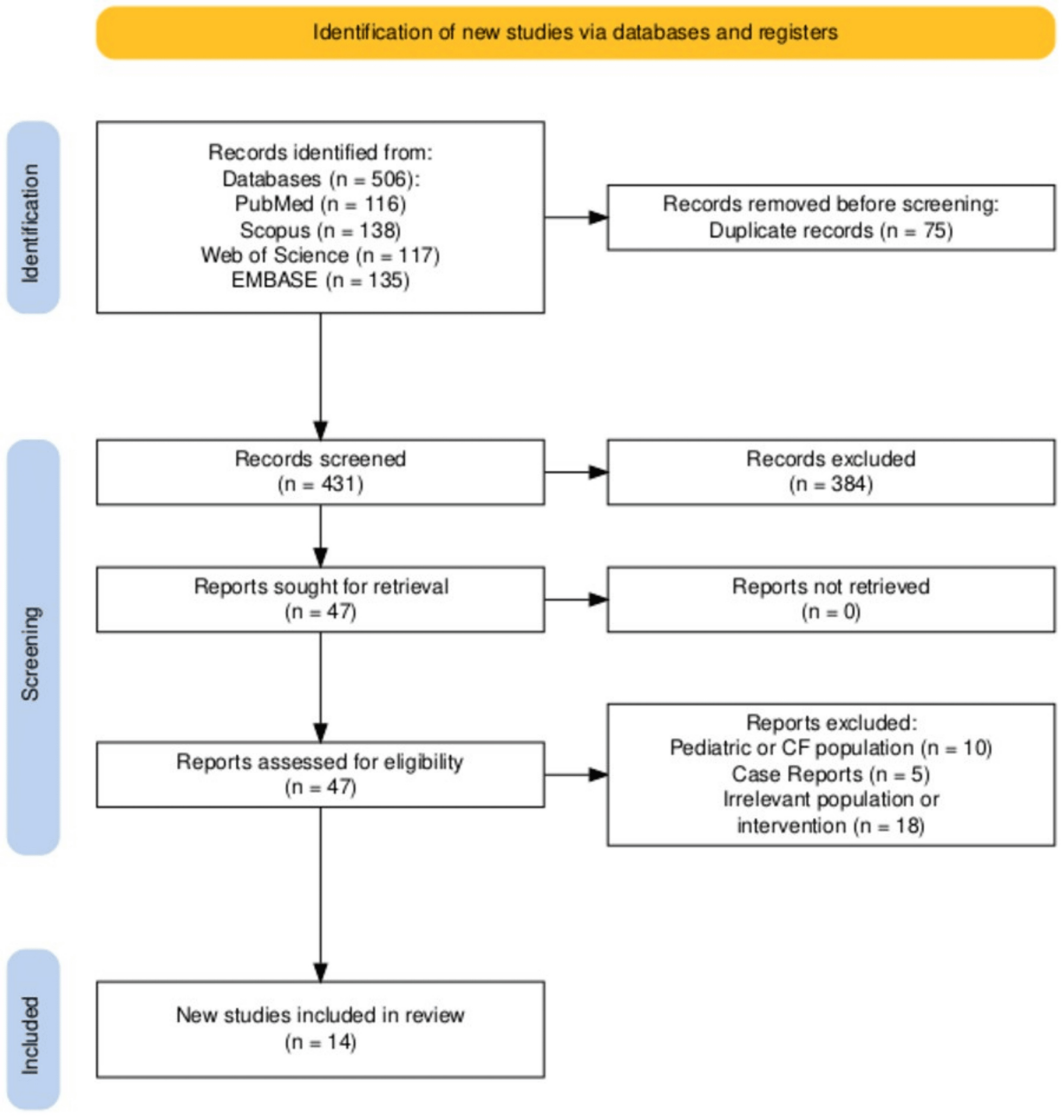
PRISMA 2020 flow diagram showing the study selection process Flow diagram depicting the identification, screening, and inclusion of studies in the systematic review in accordance with PRISMA 2020 guidelines. A total of 506 records were identified from four databases (PubMed = 116, Scopus = 138, Web of Science = 117, EMBASE = 135). After removal of 75 duplicates, 431 records were screened, and 47 full-text articles were assessed for eligibility. Twenty-three reports were excluded (10 pediatric or CF populations, five case reports, and eight irrelevant populations or interventions). Fourteen studies met the inclusion criteria and were included in the final review. PRISMA: Preferred Reporting Items for Systematic Reviews and Meta-Analyses, CF: cystic fibrosis

To contextualize local findings, a systematic review was conducted in accordance with the PRISMA 2020 guidelines. PubMed, Scopus, EMBASE, and Web of Science were searched through March 2025 using combinations of MeSH terms and free-text keywords: “*Burkholderia cepacia complex*”, “bacteremia”, “bloodstream infection”, “antimicrobial resistance”, and “clinical outcomes”. Eligible studies included observational cohorts and clinical case series reporting adult (non-CF) BCC BSIs. Two reviewers independently screened titles and abstracts, assessed full texts, and extracted data using a standardized pro forma. Risk of bias was evaluated using the NIH Quality Assessment Tool for Observational Cohort and Cross-Sectional Studies [14], with disagreements resolved by consensus. A total of 14 studies (n = 1,541 patients) met the inclusion criteria. Detailed methodological components, including the PICO (population, intervention, comparison, and outcome) framework, database-specific search strategies, and individual study risk-of-bias ratings, are provided in the Appendices. Domain-level visualizations of the NIH 14-item risk-of-bias assessment are presented in the Appendices, while the PRISMA 2020 flow diagram summarizing study selection appears in Figure 1.

Data handling

For patients with multiple BCC-positive blood cultures per admission, only the first was analyzed. Patients with incomplete clinical or microbiological data were excluded at the time of selection; therefore, all 124 included cases had complete outcome and susceptibility information.

Statistical analysis

Continuous variables were assessed for normality using the Shapiro-Wilk test. Normally distributed data were expressed as mean ± standard deviation (SD) and compared using the Student’s t-test; non-normally distributed data were summarized as median (interquartile range (IQR)) and compared using the Mann-Whitney U test. Categorical variables were presented as frequencies and compared using the chi-square test or Fisher’s exact test, as appropriate. A two-sided p < 0.05 was considered statistically significant. Multicollinearity was assessed using variance inflation factors (VIF < 2 for all predictors), and model fit was verified using the Hosmer-Lemeshow goodness-of-fit test (p = 0.64). Residual plots were visually inspected to confirm the absence of significant violations of the model. All analyses were conducted using R version 4.3.2 (R Foundation for Statistical Computing, Vienna, Austria), and figures were generated using base R functions.

## Results

Cohort characteristics

A total of 124 adult patients with culture-confirmed BCC BSI were analyzed. The median age was 48 years (IQR, 40-56), and 65 (52.4%) were male. Common comorbidities included hypertension (39.5%), diabetes mellitus (22.6%), chronic kidney disease (18.5%), and malignancy (16.1%). Overall, 33.1% of patients were immunosuppressed due to malignancy, corticosteroid therapy, or transplantation. Baseline demographic and clinical features are summarized in Table 1.

**Table 1:** Demographic and clinical characteristics of patients with BCC BSI (n = 124) * Includes malignancy, corticosteroid therapy, or transplantation. Values are presented as numbers (%) or medians (IQR). CKD: chronic kidney disease, NA: not applicable, BCC: *Burkholderia cepacia complex*, IQR: interquartile range, BSI: bloodstream infection

Characteristic	n (%)	Median (IQR)
Age (years)	NA	48 (40-56)
Male sex	65 (52.4)	NA
Smoking	35 (28.2)	NA
Alcohol use	36 (29.0)	NA
Diabetes mellitus	28 (22.6)	NA
Hypertension	49 (39.5)	NA
Chronic kidney disease	23 (18.5)	NA
Malignancy	20 (16.1)	NA
Immunosuppressive condition*	41 (33.1)	NA

Clinical course and outcomes

Nearly half of the cohort (46.8%) required ICU admission, 31.5% required mechanical ventilation, and 37.9% required vasopressor support. Organ-specific complications included AKI (33.1%), ARDS (28.2%), and MODS (19.4%). Overall in-hospital mortality rate was 33.9% (95% CI 25.7-43.2).

The prevalence of MDR infection was 64.5% (95% CI 55.8-72.4). Outcomes were numerically worse among patients with MDR infections (mortality: 38.8% vs. 25.0%; ICU admission: 52.5% vs. 36.4%), although these differences were not statistically significant (p > 0.05). Median qSOFA scores were higher among MDR cases (2 (IQR 1-2) vs. 1 (0-1)), but the difference was not significant (p = 0.79).

By contrast, comparisons according to survival status revealed marked severity gradients. Non-survivors more frequently required ICU care (78.6% vs. 30.5%, p < 0.001) and mechanical ventilation (47.6% vs. 23.2%, p = 0.010) and developed ARDS (40.5% vs. 20.7%), AKI (45.2% vs. 25.6%), and MODS (40.5% vs. 8.5%) (all p < 0.05). Median qSOFA scores were significantly higher among non-survivors (2 (IQR 2-3) vs. 1 (0-2), p < 0.001). MDR infection was slightly more frequent among those who died (73.8% vs. 59.8%), but this difference was not significant (p = 0.18). The detailed comparative analysis is presented in Table 2.

**Table 2:** Comparative clinical outcomes between MDR and non-MDR infections and between survivors and non-survivors Continuous variables are presented as median (IQR) and compared using the Mann–Whitney U test. Categorical variables are presented as n (%) and compared using the chi-square test or Fisher’s exact test when expected cell counts <5. Two-sided p < 0.05 was considered statistically significant (*). ARDS: acute respiratory distress syndrome, AKI: acute kidney injury, MODS: multiorgan dysfunction syndrome, ICU: intensive care unit, MDR: multidrug resistant, IQR: interquartile range

Variable	MDR (n = 80)	Non-MDR (n = 44)	Test statistic	Died (n = 42)	Survived (n = 82)	Test statistic
Age (years)	47 (40-57)	50 (41-55)	U = 1695.5, p = 0.371	49 (43-55)	47 (39-56)	U = 1669.5, p = 0.512
qSOFA score	2 (1-2)	1 (0-1)	U = 1375.0, p = 0.012*	2 (2-2)	1 (0-2)	U = 1184.0, p = 0.002*
ICU admission	42 (52.5%)	16 (36.4%)	χ²(1) = 2.47, p = 0.116	33 (78.6%)	25 (30.5%)	χ²(1) = 28.86, p < 0.001*
Mechanical ventilation	26 (32.5%)	13 (29.5%)	χ²(1) = 0.09, p = 0.767	20 (47.6%)	19 (23.2%)	χ²(1) = 7.52, p = 0.006*
Vasopressor use	32 (40.0%)	15 (34.1%)	χ²(1) = 0.35, p = 0.552	20 (47.6%)	27 (32.9%)	χ²(1) = 2.37, p = 0.124
ARDS	25 (31.2%)	9 (20.5%)	χ²(1) = 1.49, p = 0.222	17 (40.5%)	17 (20.7%)	χ²(1) = 5.36, p = 0.021*
AKI	27 (33.8%)	13 (29.5%)	χ²(1) = 0.19, p = 0.663	19 (45.2%)	21 (25.6%)	χ²(1) = 4.37, p = 0.037*
MODS	18 (22.5%)	6 (13.6%)	χ²(1) = 1.41, p = 0.234	17 (40.5%)	7 (8.5%)	χ²(1) = 18.77, p < 0.001*
MDR status	-	-	-	31 (73.8%)	49 (59.8%)	χ²(1) = 2.17, p = 0.141
Mortality	31 (38.8%)	11 (25.0%)	χ²(1) = 2.09, p = 0.148	-	-	-

Antimicrobial resistance profile

**Figure 2:**
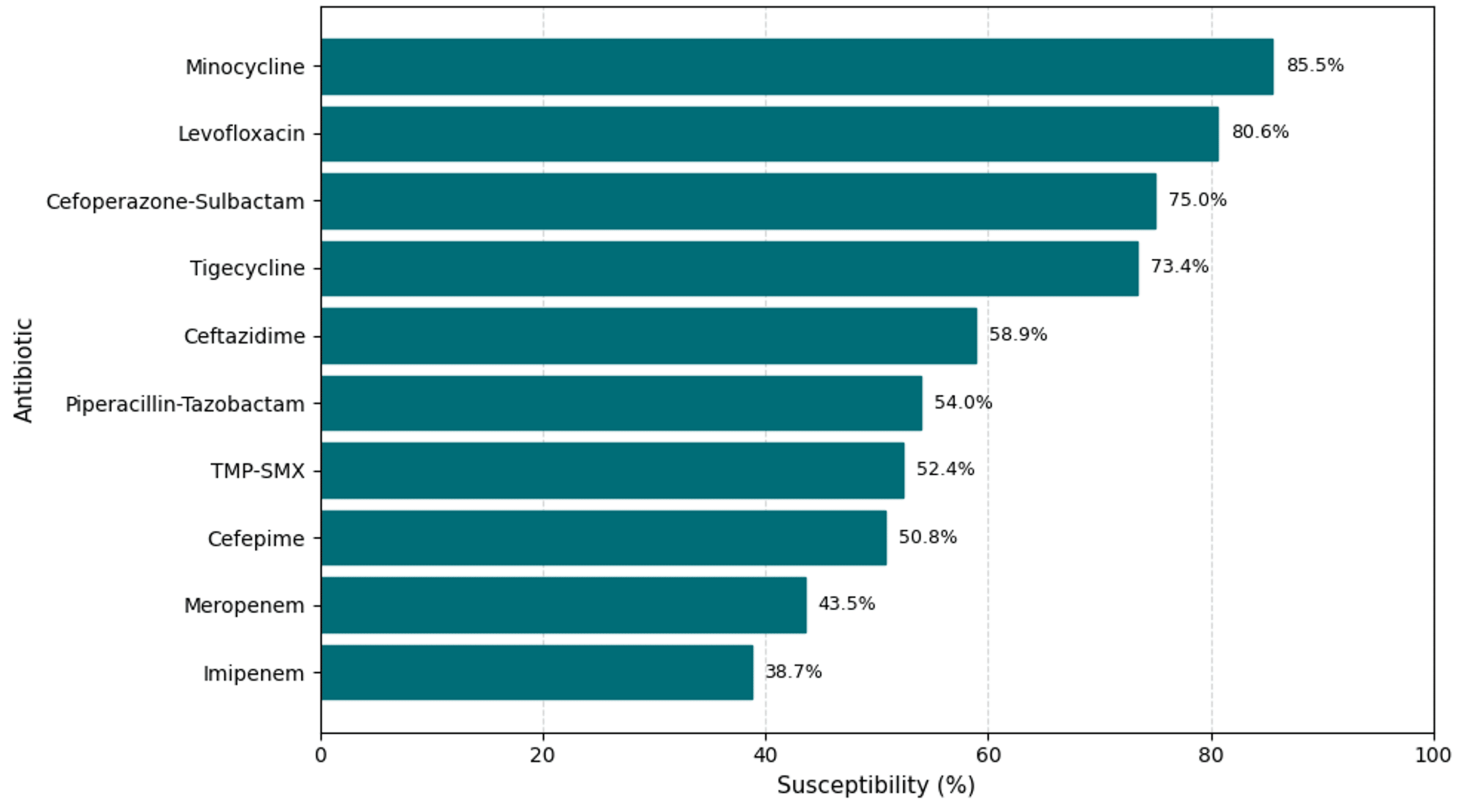
Antibiotic susceptibility profile (local cohort) Horizontal bar chart showing percentages of susceptible isolates for each tested antibiotic. Values correspond to 124 unique bloodstream isolates. TMP-SMX: trimethoprim-sulfamethoxazole

Resistance patterns of all 124 isolates are summarized in Table 3 and illustrated in Figure 2. Resistance was highest for carbapenems-imipenem (61.3%) and meropenem (56.5%), followed by cefepime (49.2%), piperacillin-tazobactam (46.0%), and TMP-SMX (47.6%). Moderate resistance was observed for ceftazidime (41.1%), tigecycline (26.6%), and cefoperazone-sulbactam (25.0%), while fluoroquinolone resistance was comparatively lower (ciprofloxacin 30.6%, levofloxacin 19.4%). Minocycline retained the most significant activity, with resistance observed in only 14.5% of isolates.

**Table 3:** Antibiotic resistance profile of BCC isolates (n = 124) Values represent the number (%) of isolates resistant to each agent. Intrinsically resistant agents (polymyxins, aminoglycosides, and aztreonam) were excluded from analysis. TMP-SMX: trimethoprim-sulfamethoxazole, BCC: *Burkholderia cepacia complex*

Antibiotic	Resistant n (%)
Imipenem	76 (61.3)
Meropenem	70 (56.5)
Cefepime	61 (49.2)
Piperacillin-tazobactam	57 (46.0)
TMP–SMX	59 (47.6)
Ceftazidime	51 (41.1)
Tigecycline	33 (26.6)
Cefoperazone-sulbactam	31 (25.0)
Ciprofloxacin	38 (30.6)
Levofloxacin	24 (19.4)
Minocycline	18 (14.5)

Intrinsic-resistance agents such as polymyxins, aminoglycosides, and aztreonam were excluded from analysis in accordance with predefined criteria. Overall, minocycline and levofloxacin exhibited the best in vitro activity, whereas resistance to carbapenems and other β-lactams exceeded 50%. The distribution of resistance percentages across all tested antibiotics is displayed in Figure 2.

Predictors of mortality

**Figure 3:**
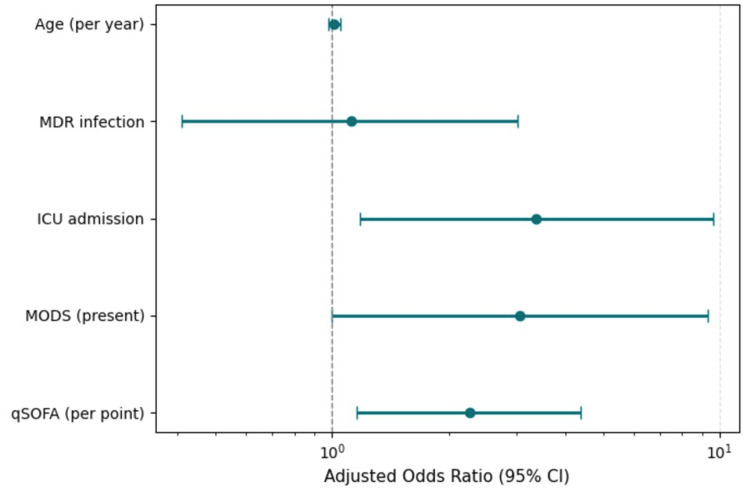
Independent predictors of mortality (multivariate logistic regression forest plot) Forest plot of adjusted ORs with 95% CIs for mortality predictors derived from logistic regression. The vertical dashed line indicates OR = 1. OR: odds ratio, CI: confidence interval, MDR: multidrug resistant, ICU: intensive care unit, MODS: multiorgan dysfunction syndrome, qSOFA: quick Sequential Organ Failure Assessment

Variables demonstrating clinical or statistical association with mortality in univariate analyses (ICU admission, MODS, AKI, qSOFA, ventilation, vasopressor use, and MDR status) were entered into multivariable logistic regression. In the final model (Table 4 and Figure 3), qSOFA, MODS, and ICU admission independently predicted in-hospital death.

**Table 4:** Multivariable logistic regression identifying independent predictors of in-hospital mortality Multivariable logistic regression analysis identifying independent predictors of in-hospital mortality among adults with BCC BSI. Data are presented as adjusted OR with 95% CI. p-values are derived from the Wald test for each model coefficient. A two-sided p < 0.05 was considered statistically significant (*). Model goodness-of-fit was assessed using the Hosmer–Lemeshow test (p = 0.64); the model explained 41% of variance (Nagelkerke R² = 0.41). OR: odds ratio, CI: confidence interval, MDR: multidrug resistant, ICU: intensive care unit, MODS: multiorgan dysfunction syndrome, qSOFA: quick Sequential Organ Failure Assessment, BCC: *Burkholderia cepacia complex*, BSI: bloodstream infection

Variable	Adjusted OR	95% CI	p-value
qSOFA (per-point increase)	2.26	1.16-4.39	0.016
MODS (present vs. absent)	3.05	1.00-9.32	0.05
ICU admission (yes vs. no)	3.37	1.18-9.60	0.023
MDR infection (yes vs. no)	1.12	0.41-3.01	0.83
Age (per year increase)	1.01	0.98-1.05	0.44

Each one-point increase in qSOFA was associated with a 2.26-fold higher odds of mortality (95% CI 1.16-4.39, p = 0.016). The presence of MODS conferred an adjusted OR of 3.05 (95% CI 1.00-9.32, p = 0.050), and ICU admission was also independently associated with death (OR 3.37 (1.18-9.60), p = 0.023). Age and MDR status were not significant predictors. The model’s Nagelkerke R² was 0.41, indicating moderate explanatory power for the observed mortality outcomes. A secondary model substituting ICU with AKI yielded similar results, confirming qSOFA and MODS as consistent mortality determinants, while MDR and age remained non-significant.

Systematic review findings

A total of 14 studies published between 2004 and 2024 were included in the systematic review, representing 1,541 adult patients with BCC BSI across North America, Europe, East and South Asia, the Middle East, and the Mediterranean region [3-6,8,15-23]. Individual cohort sizes ranged from 15 to 273 patients (median 75).

The median patient age in these studies ranged from 46 to 67 years (weighted median ≈ 58 years). Where reported, ICU admission occurred in 40% to 100% of cases, and central venous catheter (CVC) use was documented in 45% to 94% of patients, underscoring the nosocomial and device-associated nature of BCC bacteremia. Nearly all ICU-based series described mechanically ventilated or multi-organ-support patients.

In-hospital or short-term mortality varied widely, from 21% to 64%, with most cohorts reporting crude fatality between 30% and 50%. The highest mortality was observed in outbreak or transplant settings (Woods et al., Liao et al., Özdemir et al.). In contrast, larger multicenter studies such as Lee 2020 and Chang 2022 reported lower but still substantial case-fatality rates of ≈ 20-31%. Mortality generally correlated with illness severity, ICU stay, and catheter-related infection rather than study size alone.

Across studies, *Burkholderia cenocepacia* and *Burkholderia multivorans* were the most frequently identified species in cohorts performing molecular typing, while several single-center reports lacked species-level identification. MDR was commonly described, though definitions and testing methods differed considerably. Carbapenem and aminoglycoside resistance were universal themes, whereas TMP-SMX, piperacillin-tazobactam, and minocycline retained relatively high activity in most reports. Several Indian ICU studies (Baul et al., Meena et al.) linked the emergence of BCC to extensive polymyxin use, emphasizing its intrinsic colistin resistance [4,18]. Collectively, the evidence reinforces that BCC is an opportunistic, healthcare-associated pathogen predominantly affecting critically ill, device-dependent adults.

**Figure 4:**
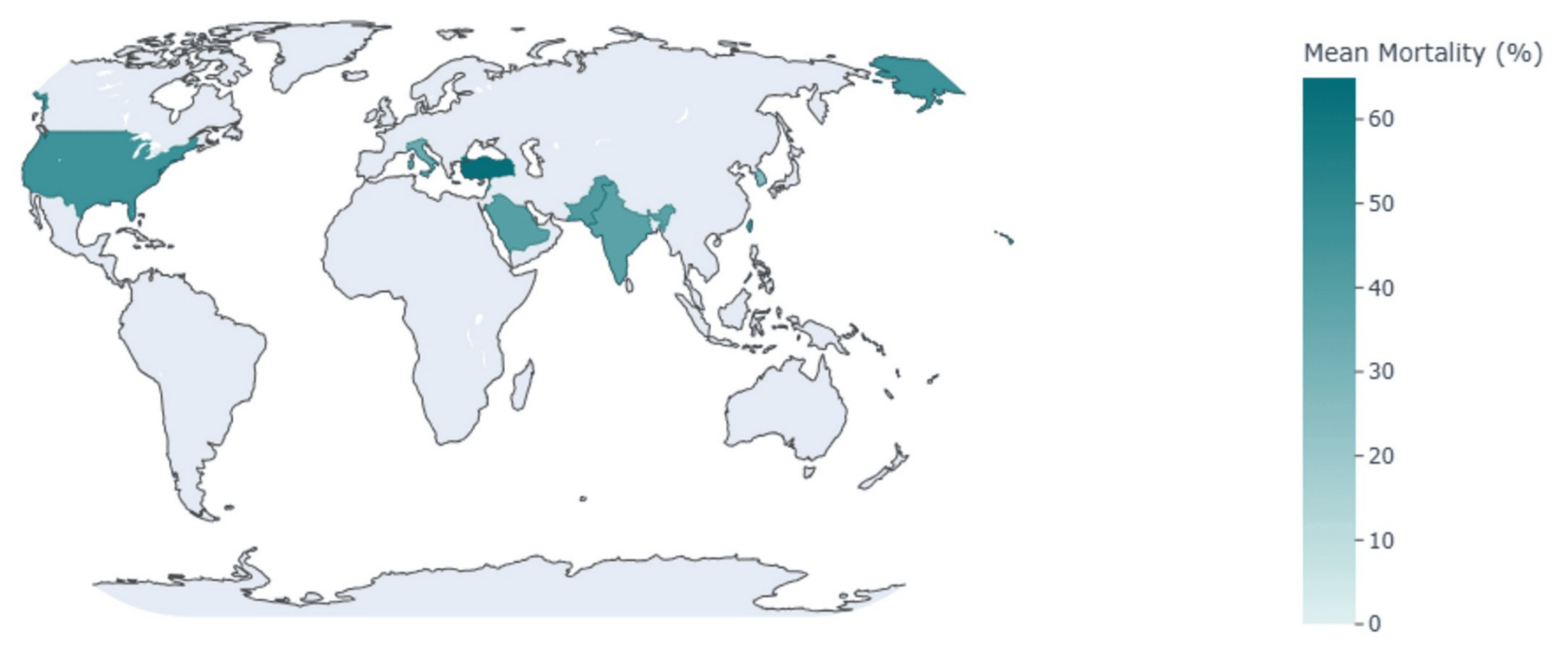
Geographic distribution of published studies and reported mortality rates Choropleth map showing the countries included in the systematic review and the mean mortality rate (%) in each region.

**Figure 5:**
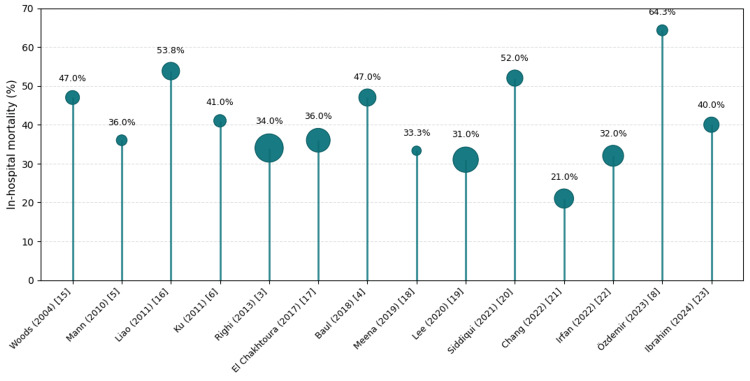
Study-wise in-hospital mortality in global literature on BCC infections Bubble (lollipop) plot of mortality rates reported in 14 published cohorts (2004–2024). Each circle represents one study; the marker area is proportional to the sample size, and vertical stems denote mortality proportion. BCC: *Burkholderia cepacia complex*

The geographic distribution of included studies and corresponding mean mortality rates is displayed in Figure 4, and inter-study variation in mortality is depicted in Figure 5. Risk-of-bias assessments using the NIH Quality Assessment Tool for Observational Cohort and Cross-Sectional Studies are summarized in the Appendices. Table 5 provides detailed study characteristics, including country, sample size, ICU exposure, CVC use, and mortality outcomes.

**Table 5:** Characteristics of studies included in the systematic review (n = 14) Mortality represents in-hospital or short-term (≤30 days) outcomes where specified. Study design/setting indicates each cohort’s methodological framework (retrospective, outbreak, multicentre, or ICU-based). ICU: intensive care unit

First author (year)	Country	Sample size (n)	Median age (years)	ICU admission (%)	Central line use (%)	Mortality (%)	Study design/setting
Woods et al. (2004) [15]	USA	53	46 (mean)	77	Not reported	47 (14-day)	Retrospective single-centre; transplant and ICU cohort
Mann et al. (2010) [5]	India	25	Not reported	≈ 60 (estimated)	Not reported	36 (in-hospital)	Retrospective case-series; tertiary ICU
Liao et al. (2011) [16]	Taiwan	95	55 (range 19-87)	100	93	53.8 (in-hospital)	Outbreak investigation; single medical ICU
Ku et al. (2011) [6]	Taiwan	39	61	58.9	94.8	41 (28-day)	Retrospective hospital cohort
Righi et al. (2013) [3]	Italy	273	59	38.1	Not reported	34 (in-hospital)	Multicentre observational cohort
El Chakhtoura et al. (2017) [17]	Lebanon / USA	188	55 (mean)	≈ 40 (HAI subset)	Not reported	36 (90-day)	Multicentre retrospective cohort
Baul et al. (2018) [4]	India	90	57	55	50	47 (in-hospital)	Retrospective ICU cohort; tertiary hospital
Meena et al. (2019) [18]	India	15	51.8 (mean)	100	Not reported	33.3 (in-hospital)	ICU emergence report; single centre
Lee et al. (2020) [19]	South Korea	216	60	43 (ICU stay)	56.9 (CVC present)	31 (in-hospital)	Multicentre retrospective cohort
Siddiqui et al. (2021) [20]	Pakistan	78	64	66.7	71.8	52 (in-hospital)	Single-centre retrospective cohort
Chang et al. (2022) [21]	South Korea	117	67	43.6	Not reported	21 (in-hospital)	Multicentre hospital cohort
Irfan et al. (2022) [22]	Pakistan	142	59	40.1	Not reported	32 (in-hospital)	Retrospective observational study
Özdemir et al. (2023) [8]	Turkey	27	62	55.5	65	64.3 (in-hospital)	Single-centre ICU cohort; haematology population
Ibrahim et al. (2024) [23]	Saudi Arabia	68	52	41.2	45.6	40 (in-hospital)	Retrospective tertiary-care cohort

Across the 14 eligible studies included in the PRISMA-guided systematic review, there was qualitative heterogeneity in design, population, and MDR definitions. Formal meta-analysis and I² estimation were not performed due to these methodological differences; instead, heterogeneity was assessed qualitatively and discussed narratively in the Discussion section.

## Discussion

Principal findings

In this retrospective cohort of adults with BCC BSI, the in-hospital mortality rate was 33.9%, and 64.5% of isolates met criteria for MDR. Carbapenem resistance was common (imipenem 61.3%, meropenem 56.5%), whereas minocycline (85.5% susceptible) and levofloxacin (80.6% susceptible) retained the highest in vitro activity (Figure 2). In multivariable analysis, higher qSOFA score, intensive care admission, and multiorgan dysfunction independently predicted mortality, whereas MDR phenotype and age were not significant. The lack of independent association between MDR status and mortality may reflect survival bias (critically ill patients dying before culture confirmation) or timely empiric therapy mitigating the effect of resistance. These findings suggest that illness severity rather than resistance pattern was the principal determinant of outcome.

The accompanying systematic review of 14 studies (2004-2024; n = 1,541) found comparable results, with mortality rates ranging from 21% to 64% and most clustering around 30-50%. BCC infections were consistently described as nosocomial, device-associated, and affecting critically ill adults, supporting the observations from our local cohort.

These results have critical infection-control implications for low- and middle-income hospital settings, where resource constraints and device reuse heighten transmission risk. Reinforcing hand hygiene, ensuring single-use or appropriately sterilized catheters and ventilator circuits, and implementing routine BCC bloodstream-infection surveillance could substantially reduce the potential for outbreaks.

Comparison with regional and global studies

Our results align closely with contemporary data from Asia and the Middle East. In Pakistan, Irfan et al. [22] reported a 32% in-hospital mortality and extensive carbapenem resistance, while Siddiqui et al. [20] documented 52% mortality and high ICU prevalence. In Saudi Arabia, Ibrahim et al. [23] observed 40% mortality among non-CF adults, with the best activity for ceftazidime and TMP-SMX. Indian ICU reports similarly describe high resistance to β-lactams but retained susceptibility to minocycline and fluoroquinolones [4,18].

Large multicenter cohorts from East Asia, including Lee et al. (Korea) and Chang et al. (Korea), reported lower but still substantial mortality (≈ 20-31%) and identified septic shock, liver disease, and catheter-related bacteremia as major predictors of death [19,21]. ICU outbreaks in Taiwan and the United States showed that BCC frequently colonizes central-line circuits and aqueous reservoirs, with mortality exceeding 50% during outbreak phases [6,15,16]. Similar nosocomial patterns were reported in Italy, Lebanon/USA, Turkey, and India [3,5,8,17]. Collectively, these data confirm the global entrenchment of BCC as a hospital-adapted pathogen across both high- and middle-income settings.

Across studies, TMP-SMX and piperacillin-tazobactam remain the most reliable agents, typically active in ≥85-90% of isolates [17,19,23]. Minocycline consistently demonstrates >90% susceptibility [4,18,19], paralleling our findings. Ceftazidime shows variable activity (60-80%), whereas carbapenem susceptibility has declined markedly, especially in ICU environments [16,18,22]. Fluoroquinolone activity ranges from 60% to 85% [18,23]. All studies reaffirm intrinsic resistance to polymyxins, aminoglycosides, and aztreonam [17].

Clinical management implications

Given this resistance profile, empiric carbapenem therapy is unreliable when BCC is suspected. Minocycline and TMP-SMX demonstrated the highest retained activity in our cohort (Table 3, Figure 2) and are supported by global data [4,17-19,23]. These agents may be appropriate empiric options in high-risk nosocomial settings, pending susceptibility confirmation.

Combination therapy is commonly used for severe infections but lacks a proven survival benefit. Lee et al. [19] found no outcome difference between TMP-SMX, ceftazidime, or carbapenem monotherapy, and Siddiqui et al. [20] similarly reported no advantage for combination regimens. In our cohort, MDR status itself did not independently predict mortality, reinforcing that timely therapy and host factors outweigh the resistance phenotype.

Source control remains pivotal. Early catheter removal in suspected line-associated BCC bacteremia improves microbiologic clearance [3,15,19], and strict environmental decontamination is essential because the organism persists in moist reservoirs [16]. Strengthened ICU surveillance, device sterilization practice audits, and reinforcement of staff hand hygiene are practical priorities for infection-control programs in resource-limited hospitals.

Species-level diagnostics and resistance testing

Species-level identification was not performed locally, limiting direct comparisons across genomovars. Prior studies show that *Burkholderia cenocepacia* is particularly virulent and transmissible [15,17]. Future work should incorporate molecular species-level identification using updated MALDI-TOF libraries or recA sequencing to differentiate *Burkholderia cenocepacia*, *Burkholderia multivorans*, and related species, enabling genotype-phenotype correlation and targeted infection-control measures. While CLSI M100 (33rd edition) [10] remains the interpretive standard, many emerging agents, such as minocycline and cefiderocol, lack genus-specific breakpoints, complicating inter-laboratory comparison.

Strengths and limitations

This study integrates a longitudinal single-center cohort with a comprehensive systematic review, offering both local and global context. We applied standardized MDR definitions [11], restricted susceptibility testing to clinically relevant agents, and excluded intrinsically inactive antibiotics to avoid overestimating resistance.

Limitations include a retrospective design, the absence of species-level data, and potential misclassification arising from treating intermediate results as susceptible. Data on time to appropriate therapy, pharmacodynamic optimization, and duration of organ support were unavailable. The systematic review was descriptive, as heterogeneity in definitions and reporting precluded formal meta-analysis (Table 5).

## Conclusions

In this cohort of adults with BCC BSI, illness severity rather than MDR was the primary determinant of in-hospital mortality. High rates of resistance to β-lactams and carbapenems substantially limit empiric options, while minocycline, TMP-SMX, and levofloxacin remain the most consistently active agents.

When viewed alongside global data from 2004 to 2024, these findings reaffirm BCC as an opportunistic, nosocomial pathogen affecting critically ill and device-dependent patients worldwide. Key priorities include adopting rapid molecular or MALDI-TOF-based species identification, strengthening antimicrobial-stewardship programs to ensure timely, appropriate therapy, and promptly implementing source control to prevent relapse or persistent infection.

Local resistance trends should continue to guide empiric therapy, particularly in intensive-care settings where BCC often persists in environmental reservoirs. Future research should focus on species-specific outcomes, molecular mechanisms of resistance, and the role of newer agents, such as cefiderocol and optimized tetracycline combinations, in MDR BCC.

## Figures and Tables

**Figure 6:**
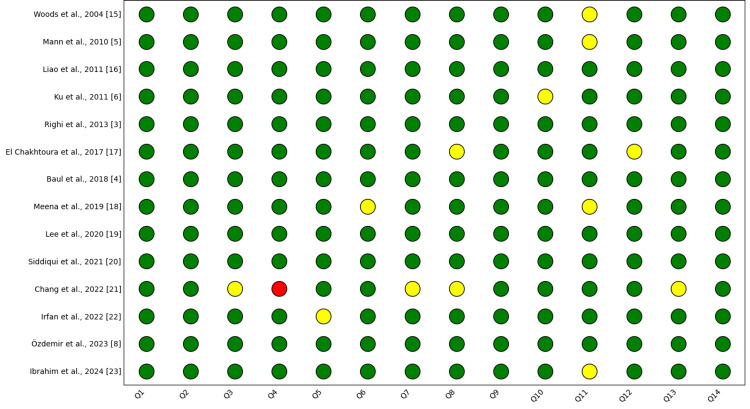
Risk of bias traffic light plot This figure visually summarizes the risk of bias assessment across all included studies using the NIH Quality Assessment Tool for Observational Cohort and Cross-Sectional Studies. Each row corresponds to a study, and each column represents one of the fourteen methodological domains evaluated. The color of each circle indicates the judgment assigned for that domain: green represents “yes” (criterion met, indicating low risk), red represents “no” (criterion not met, indicating high risk), and yellow represents “cannot determine” or “not applicable” (uncertain or inapplicable assessment). The domains Q1 through Q14 cover key aspects of study quality, including clarity of objectives, population definition, sample size justification, timing of exposure and outcome measurement, outcome blinding, follow-up adequacy, and control of confounding. Detailed descriptions of each domain are available in the Materials and Methods and Appendices. NIH: National Institutes of Health

**Figure 7:**
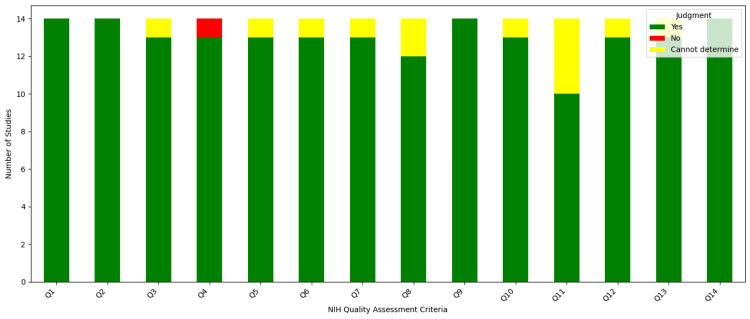
Risk of bias summary bar plot This figure presents a domain-wise summary of risk-of-bias judgments across all included studies using the NIH 14-item assessment tool. Each bar represents one domain (Q1 to Q14) and shows the number of studies that received a “yes” (green), “no” (red), or “cannot determine” (yellow) rating for that specific criterion. The plot provides an at-a-glance overview of methodological strengths and uncertainties across the evidence base. NIH: National Institutes of Health

## Appendices

Supplementary material: search strategy and risk of bias assessment

Overview

This supplementary file provides additional methodological information on the systematic review conducted for the present study. It includes the research question, the PICO framework, a database-specific search strategy, details of the risk-of-bias assessment, and visual representations of quality appraisal using ROBVIS. The PRISMA 2020 flow diagram depicting study selection is provided in the main manuscript as Figure 1. All screening, extraction, and quality assessments were independently conducted by two reviewers, with disagreements resolved through consensus.

Research Question

The systematic review addressed the following research question: What are the clinical profiles, antimicrobial resistance patterns, and mortality outcomes associated with BCC infections in adult, non-cystic fibrosis patients, and what prognostic factors have been identified in published literature? The review was structured using the PICO framework, summarized in Table 6.

**Table 6:** PICO framework BCC: *Burkholderia cepacia complex*, ICU: intensive care unit, PICO: population, intervention, comparison, and outcome

Component	Description
Population	Adult patients (≥18 years) with confirmed BCC infections, excluding cystic fibrosis patients
Intervention/exposure	Infection with BCC, including bloodstream or severe nosocomial infections
Comparator	Patients without BCC infection or with different resistance profiles, depending on study design
Outcomes	In-hospital mortality, ICU admission, recurrence, multidrug resistance, and associated prognostic factors

Databases and Search Queries Used in the Systematic Review

A comprehensive literature search will be conducted across PubMed, Scopus, Web of Science, and EMBASE to identify relevant studies on *Burkholderia cepacia* infections, clinical outcomes, and antimicrobial resistance. The search will be conducted up to March 2025, with no restriction on publication year. The search strategy combines MeSH terms and free-text keywords to ensure comprehensive retrieval of global evidence and is provided in Table 7.

**Table 7:** Search strategy for each database

Database	Search query
PubMed	(("Burkholderia cepacia"[MeSH Terms] OR "Burkholderia cepacia" OR "B. cepacia") AND ("Bacteremia" OR "Bloodstream infection" OR "Sepsis") AND ("Mortality" OR "ICU admission" OR "ARDS" OR "MODS" OR "Outcome") AND ("Antibiotic resistance" OR "Multidrug resistance" OR "MDR" OR "Resistance pattern" OR "Susceptibility")) AND (("Case Reports"[Publication Type] OR "Observational Study"[Publication Type] OR "Retrospective Study"[Publication Type] OR "Clinical Study"[Publication Type] OR "Review"[Publication Type] OR "Systematic Review"[Publication Type]))
Scopus	TITLE-ABS-KEY("Burkholderia cepacia" OR "B. cepacia") AND TITLE-ABS-KEY("Bacteremia" OR "Bloodstream infection" OR "Sepsis") AND TITLE-ABS-KEY("Mortality" OR "Outcome" OR "ICU" OR "ARDS" OR "MODS") AND TITLE-ABS-KEY("Antibiotic resistance" OR "MDR" OR "Resistance pattern") AND (LIMIT-TO(DOCTYPE, "ar")) AND (LIMIT-TO(LANGUAGE, "English"))
Web of Science	TS=("Burkholderia cepacia") AND TS=("bacteremia" OR "sepsis" OR "bloodstream infection") AND TS=("mortality" OR "ICU admission" OR "ARDS" OR "MODS") AND TS=("antibiotic resistance" OR "MDR") AND DT=(Article OR Case Report) AND LA=(English)
EMBASE	'burkholderia cepacia'/exp OR 'burkholderia cepacia':ti,ab AND ('bacteremia'/exp OR 'sepsis':ti,ab OR 'bloodstream infection':ti,ab) AND ('mortality'/exp OR 'ICU admission':ti,ab OR 'ARDS':ti,ab OR 'MODS':ti,ab) AND ('multidrug resistance'/exp OR 'antibiotic resistance':ti,ab) AND [article]/lim AND [english]/lim

NIH 14-Item Risk of Bias Tool Description (Narrative Format)

The NIH Quality Assessment Tool for Observational Cohort and Cross-Sectional Studies evaluates internal validity across fourteen methodological domains. These domains assess the clarity of the research question, the definition of the study population, and the adequacy of the participation rate. It further examines whether participants were drawn from comparable populations and whether sample size justifications, power estimates, or variance measures were provided.

The tool also evaluates whether exposures were measured before outcomes and whether the duration of follow-up was sufficient to observe the association. In studies involving exposures with varying levels, it assesses whether such variations were appropriately examined. Additionally, it reviews whether exposure and outcome measures were clearly defined, valid, reliable, and implemented consistently across participants.

Further attention is given to assessment processes, including blinding outcome assessors to exposure status and ensuring adequate follow-up. Finally, the tool evaluates whether potential confounding variables were appropriately measured and statistically adjusted for in the analysis.

Each included study was evaluated against these domains and scored as “yes,” “no,” “cannot determine,” or “not applicable.” An overall risk of bias rating was determined based on the number of “yes” responses. Studies with eleven or more “yes” responses were classified as low risk, those with eight to ten as moderate risk, and those with fewer than eight as high risk. The final overall scores for each study are summarized in Table 8, while domain-specific judgments are visually presented in Figure 6 (traffic light plot) and Figure 7 (summary bar plot).

**Table 8:** Overall NIH risk of bias scores for included studies Risk of bias was assessed using the NIH Quality Assessment Tool for Observational Cohort and Cross-Sectional Studies. Each study was scored based on the number of domains marked “yes” out of 14 possible criteria. Studies with 11 or more “yes” responses were rated as low risk, those with 8 to 10 as moderate risk, and those with fewer than 8 as high risk. NIH: National Institutes of Health

Study (author, year)	NIH score (out of 14)	Overall risk of bias
Woods et al., 2004 [15]	9	Moderate risk
Mann et al., 2010 [5]	9	Moderate risk
Liao et al., 2011 [16]	12	Low risk
Ku et al., 2011 [6]	12	Low risk
Righi et al., 2013 [3]	12	Low risk
El Chakhtoura et al., 2017 [17]	11	Low risk
Baul et al., 2018 [4]	12	Low risk
Meena et al., 2019 [18]	11	Low risk
Lee et al., 2020 [19]	11	Low risk
Siddiqui et al., 2021 [20]	11	Low risk
Chang et al., 2022 [21]	9	Moderate risk
Irfan et al., 2022 [22]	10	Moderate risk
Özdemir et al., 2023 [8]	13	Low risk
Ibrahim et al., 2024 [23]	12	Low risk
